# A String of Pearls from the *Post-Graduate* for April, 1891

**Published:** 1891-05

**Authors:** 


### A STRING OF PEARLS FROM THE POST-GRADUATE FOR APRIL, 1891.

We take pleasure in presenting to our readers this group of editorial paragraphs from the *Post-Graduate*, which we are sure will prove of interest :

The signs of the times for medical science are promising. In the State of New York we now have a compulsory three years' course, prefaced by a compulsory preliminary examination, and ended by an independent examination for a license to practise before a Board of Medical Examiners appointed by the State. Certainly we are having legislation enough to get on.

The State Medical Society was a noble gathering, probably one of the largest that ever has been held. Dr. Reed, of Cincinnati, at the dinner at the Delavan House, in view of the feeble condition and small numbers of the Ohio State Medical Society, suggested that he had better go home and get up a secession, so that it might be invigorated as the New York Society seems to have been. Indeed, the secession of 1882 does not seem to have decreased the numbers or diminished the enthusiasm of the Society that was founded in 1807, and has not failed to have a meeting every year since. New men are coming in fast, to fill the places of those who have passed away, or who have become old in the service. The scientific work will compare favorably with that of any society in the country, while the social element predominates very strongly. Dinners, public and private, are given, beginning on Monday night, until the close of the session. The Albanians, with their proverbial hospitality, do everything possible to abate the rigor of the Northern winter and the bad quality of their drinking water, during the three days that the Society is in session in the ancient capital. Do all they can, the winter is always bad when the Society meets, but it makes a good break in it, and the countrymen as well as the New Yorkers seem to enjoy the dissipation exceedingly.

The most important work of the Society, in a political sense, was the nomination of fourteen men, from whom the Regents should select seven to be examiners in medicine and surgery. For, after this, unless the law is repealed, no man can practise medicine in the State of New York, except those already in the profession, unless he shall have received a license from this Board.

New York City has two very interesting and ably conducted weekly medical journals, but although the greatest medical event that has occurred in the State for many a year was consummated at Albany, when fourteen members of the profession were named to the Board of Regents from whom to select seven examiners who should determine all those to whom a license to practise medicine in the State should be granted, so far as we have observed, no leader or editorial paragraph as to the final triumph of the reform—for which one of them has often shrieked itself hoarse—was to be seen the week after the Medical Society adjourned, or the week after that.

And now that we are on that subject, it will relieve us to say that, although medical bills are being presented to the Legislature almost every week—bills affecting the interest of the medical profession, and through them of the public throughout the State—the publishers of these journals do not seem to be willing to pay the few dollars necessary to send a record to the medical profession of what is going on. We cannot imagine it is the fault of the editors; they certainly must know what a valuable contribution this would be to the medical news of the day.

Perhaps the *Record* said nothing about the fourteen names because one of them was that of the editor of that journal. Certainly, however, one of the staff might have ventured to make a few comments on the others.

Reforms are born with many throes, while abuses and evil systems die hard. Already a bill has been introduced into the Legislature, to practically annul the new law for three years, and all this to allow some youths who began the study of medicine before June, 1890, to pass their examination in the good old way before the college faculty. If there was no need for an independent examination, why this solicitude to pass only the faculty examinations? Poor tender youths, the profession can readily spare them! If we except them, why not also those who began before the year 2000, and so on? Is there any particular virtue in any young man who began the study of medicine before 1890, that he should be exempted before the provisions of the law that the State Legislature had been almost fifteen years in passing?

If we do not mistake the temper of the Legislature, they will not tinker with this State Examination Law this year, but will allow the people to give it a trial before it is amended or repealed. They have heard too many committees and too much talk in the last ten years, not to know that the subject of a State Examining Board is exhausted, for a time at least, and that a rest from the whole subject is demanded until we know what legislation has accomplished.

